# Target temperature management following cardiac arrest: a systematic review and Bayesian meta-analysis

**DOI:** 10.1186/s13054-022-03935-z

**Published:** 2022-03-12

**Authors:** Anders Aneman, Steven Frost, Michael Parr, Markus B. Skrifvars

**Affiliations:** 1 Intensive Care Unit , Liverpool Hospital, South Western Sydney Local Health District, Locked Bag 7103, Liverpool BC, NSW 1871 Australia; 2 South Western Clinical School, University of New South Wales, Sydney, NSW Australia; 3 Faculty of Health Sciences, Macquarie University, Sydney, NSW Australia; 4 Centre for Applied Nursing Research, Ingham Institute for Applied Medical Research, University of New South Wales, Sydney, NSW Australia; 5 Department of Emergency Care and Services, Helsinki University Hospital and University of Helsinki, Helsinki, Finland; 6 University of Helsinki, Helsinki, Finland

**Keywords:** Cardiac arrest, Target temperature management, Bayesian statistics

## Abstract

**Background:**

Temperature control with target temperature management (TTM) after cardiac arrest has been endorsed by expert societies and adopted in international clinical practice guidelines but recent evidence challenges the use of hypothermic TTM.

**Methods:**

Systematic review and Bayesian meta-analysis of clinical trials on adult survivors from cardiac arrest undergoing TTM for at least 12 h comparing TTM versus no TTM or with a separation > 2 °C between intervention and control groups using the PubMed/MEDLINE, EMBASE, CENTRAL databases from inception to 1 September 2021 (PROSPERO CRD42021248140). All randomised and quasi-randomised controlled trials were considered. The risk ratio and 95% confidence interval for death (primary outcome) and unfavourable neurological recovery (secondary outcome) were captured using the original study definitions censored up to 180 days after cardiac arrest. Bias was assessed using the updated Cochrane risk-of-bias for randomised trials tool and certainty of evidence assessed using the Grading of Recommendation Assessment, Development and Evaluation methodology. A hierarchical robust Bayesian model-averaged meta-analysis was performed using both minimally informative and data-driven priors and reported by mean risk ratio (RR) and its 95% credible interval (95% CrI).

**Results:**

In seven studies (three low bias, three intermediate bias, one high bias, very low to low certainty) recruiting 3792 patients the RR by TTM 32–34 °C was 0.95 [95% CrI 0.78—1.09] for death and RR 0.93 [95% CrI 0.84—1.02] for unfavourable neurological outcome. The posterior probability for no benefit (RR ≥ 1) by TTM 32–34 °C was 24% for death and 12% for unfavourable neurological outcome. The posterior probabilities for favourable treatment effects of TTM 32–34 °C were the highest for an absolute risk reduction of 2–4% for death (28–53% chance) and unfavourable neurological outcome (63–78% chance). Excluding four studies without active avoidance of fever in the control arm reduced the probability to achieve an absolute risk reduction > 2% for death or unfavourable neurological outcome to ≤ 50%.

**Conclusions:**

The posterior probability distributions did not support the use of TTM at 32–34 °C compared to 36 °C also including active control of fever to reduce the risk of death and unfavourable neurological outcome at 90–180 days. Any likely benefit of hypothermic TTM is smaller than targeted in RCTs to date.

**Supplementary Information:**

The online version contains supplementary material available at 10.1186/s13054-022-03935-z.

## Background

Temperature control with target temperature management (TTM) after cardiac arrest has been endorsed by expert societies and adopted in international clinical practice guidelines [1–3] based on several randomised controlled trials (RCTs), systematic reviews and meta-analyses [4–10]. The original studies and systematic reviews have exclusively used frequentist methods for statistical inference. A growing body of literature is instead using Bayesian statistics to interpret the results of clinical trials, i.e. the likelihood, in the context of already existing beliefs on treatment effects, i.e. the prior, that when combined generate a posterior distribution of probabilities for the effect size [11–13]. This brings the trial effect estimates beyond the dichotomous outcome as being significant or non-significant based on a *p* value of 0.05 in frequentist hypothesis testing and instead attributes probabilities to effect sizes contained in the 95% credible interval. Bayesian analyses have been proposed to supplement interpretations of clinical trials in critical care [14], to inform clinical practice guidelines in cardiology [15] and the US Food and Drug Administration has issued guidelines for their use in clinical trials of medical devices [16].

The recently published trial by Dankiewicz et al. [17], the largest study performed to date on the use of TTM in post-cardiac arrest care, compared the institution of TTM at 33–34 °C for 24 h with active measures to maintain normothermia (< 37.5 °C) if the body temperature increased above 37.8 °C. The study was powered to detect an absolute risk reduction for death by 7.5% by frequentist inference [18, 19] and did not observe any benefit of hypothermic TTM on all-cause mortality or poor functional recovery. The aim of this review and Bayesian meta-analysis was to evaluate the effect of TTM on survival and neurological outcome compared to no TTM or avoiding pyrexia in the care of adult, comatose survivors of cardiac arrest. The null hypothesis of no difference between patients treated with TTM at 32–34 °C or TTM ≥ 36 °C was compared to the alternative hypothesis that hypothermic TTM confers a benefit using a range of data-informed priors to reflect optimism, pessimism or equipoise regarding the effect of TTM. By using Bayesian statistical inference, the posterior probabilities attributed to the absence of any benefit could be compared to a range of treatment effects, including ones smaller than targeted in the original studies.

## Methods

This review and meta-analysis were performed in accordance with the protocol registered with PROSPERO (CRD42021248140) and are reported according to the PRISMA statement [20] (Additional File 1: Table S1) and the ROBUST criteria [21] (Additional File 1: Table S2).

### Eligibility criteria

All randomised and quasi-randomised (e.g. allocation based on the day of week) controlled trials of adult (≥ 18 years of age) comatose survivors from cardiac arrest undergoing TTM for at least 12 h were included. Hence, both in-hospital (IHCA) and out-of-hospital cardiac arrest (OHCA) with all initial rhythms and any locations to initiate TTM were considered. A separation between intervention and control groups in TTM studies was accepted as any TTM temperature compared to no TTM or a difference > 2 °C in target temperature between groups. The intervention and control groups are referred to by the target temperature, i.e. TTM_32–34_ versus TTM_≥36_. We excluded studies comparing similar TTM but of different duration, investigating hypothermic (32–34 °C) TTM in both interventional and control arms, using different devices to induce TTM or studies focusing on the setting of TTM, e.g. pre-hospital versus in-hospital studies.

### Information sources and search strategy

The PubMed/MEDLINE, EMBASE, CENTRAL bibliographic databases and the clinicaltrials.gov trial database were searched using a search strategy directed towards randomised controlled trials in human adults from inception to the 1 September 2021 (original search, Additional File 1: Table S3) with an expanded search performed during editorial review (last updated 24 January 2022, Additional File 1: Table S4). Search results were uploaded to Covidence (Veritas Health Innovation, Melbourne, Australia). Only studies providing the essential data were considered. Titles and abstracts were screened for potential eligibility with the full text reviewed in case of unclear potential. Citations from included articles were also reviewed as were citations in published systematic reviews and meta-analyses.

### Data items collected, risk of bias and certainty of evidence

A standardised data abstraction form was used to capture author, publication year, cardiac arrest characteristics, details of temperature management in control and intervention groups, the number of events and patients in in each group and times when outcomes were censored. Survival (primary outcome) and neurological status (secondary outcome) using the definitions in the original studies were censored within up to 180 days after cardiac arrest. Most neurocognitive recovery occurs within the first 90–180 days timeframe [22, 23] that has commonly been used in RCTs of TTM. Short-term neurological outcome, e.g. at discharge from intensive care or from hospital, might be susceptible to confounding by the time of discharge reflecting the speed and quality of recovery following cardiac arrest. These timeframes are reported separately in this review. Domains of bias were assessed using the updated Cochrane risk-of-bias (RoB 2) for randomised trials tool [24] and certainty of evidence assessed using the Grading of Recommendation Assessment, Development and Evaluation (GRADE) methodology [25]. The search, study selection, data extraction, bias and GRADE assessments were performed independently by two authors (AA and MS), and any discrepancies were resolved by consensus involving a third researcher (SF).

### Effect measures, data synthesis and sensitivity analyses

The risk ratio (RR) and its upper and lower 95% confidence interval (95% CI) for death and unfavourable neurological outcome were retrieved or calculated for each study. Bayesian meta-analysis was used to examine the studies in aggregate and sequentially, i.e. using incremental evidence to generate posterior probabilities for the fixed-effect and random-effect models [26] of the null hypothesis (H_0_, there is no difference between TTM_32–34_ and TTM_≥36_, illustrated by the Bayes factor BF_01_, a likelihood ratio in favour of H_0_) and the alternative hypothesis (H_1_, TTM_32–34_ results in a difference compared to TTM_≥36_, illustrated by the Bayes factor BF_10_, a likelihood ratio in favour of H_1_). The likelihood ratio contained in the Bayes factor (BF) represents a metric for the strength of supporting evidence. A hierarchical robust Bayesian model-averaged meta-analysis [27] was performed that combined the results of Bayesian fixed-effect and Bayesian random-effect models to generate a model average for effect size, heterogeneity and publication bias [28]. A range of priors were set for the effect size with the mean representing the belief where the treatment effect is centred (RR < 1, TTM_32–34_ confers benefit; RR = 1, no effect; RR ≥ 1 TTM_32–34_ does not confer benefit) and the variance (standard deviation) representing the certainty in the belief (a certain belief has a narrower variance compared to an uncertain belief) (Additional File 1: Table S4). A minimally informative effect size prior was set as RR = 1 (no effect of intervention) with a Cauchy distribution of probabilities (i.e. a continuous distribution of probabilities) meaning that all information is provided by the studies. The minimally informed prior hence produces results most similar to a frequentist meta-analysis. In addition, effect size priors informed by meta-analysis or reflecting a range from strongly enthusiastic to strongly pessimistic beliefs about plausible TTM outcomes were used [29]. Frequentist meta-analysis of binary outcomes by random effects estimates using the DerSimonian–Laird method was performed to generate informed priors (Additional File 1: Fig. S1). Data-driven priors for between-study heterogeneity [30] were used together with priors for publication bias. All prior settings are reported in Table 1). The model-average overall effect is reported as the mean RR including its 95% credible interval (95% CrI), i.e. the interval within which there is a 95% probability the treatment effect resides. The Markov chain Monte Carlo (MCMC) algorithm (3 chains, 10,000 iterations, burn-in of 5000 iterations, adaptation of 1000 iterations, thinning of 1, target margin of error 1%, target Rhat < 1.05, exclude models with Rhat > 1.05) was used to derive posterior effect estimates and 95% CrIs. Convergence of the MCMC chains was assessed by Rhat at 1, visual inspection of the trace and density plots to confirm that the chains were producing representative values from the posterior distribution. Finally, the autocorrelation plots were inspected to demonstrate essentially zero autocorrelation. Various treatment effects of TTM were evaluated based on the posterior probabilities for the overall effect size. First, considering the frequentist approach to refute the null hypothesis (H_0_), any decrease in mortality (RR < 1) was considered. Second, an absolute risk reduction (ARR) in mortality by 7.5% was explored as per the most recent trial protocol [18, 19]. Third, the incidence of OHCA in the US [31] and Europe [32] provide a crude theoretical estimate of 300,000 OHCA patients annually meaning that a significantly smaller ARR of 2% would still translate into 6000 lives saved every year and arguably represents a minimum clinically important difference [33]. Fourth, a range of ARR at 4%, 6% and 10% was included to illustrate treatment effects below and above the protocol effect size estimate. The baseline was set using data from the International Cardiac Arrest Registry and a separation of TTM aligned with this review [34]. Fifth, the posterior probability for no benefit of TTM was assessed by a RR ≥ 1. Sensitivity analyses were performed for studies that only reported short-term neurological outcome, did or did not apply an explicit temperature definition of normothermia and the avoidance of fever in the control groups and for studies that reported patients with initial shockable versus non-shockable rhythms separately.

**Table 1:** Settings of the data-driven effect size, study heterogeneity and publication bias priors for the primary outcome mortality and secondary outcome unfavourable neurology

Prior description	Risk ratio, mean	Risk ratio, standard deviation
Death		
Minimally informative	Cauchy distribution with location = 1	NA
Informed based on frequentist random effects meta-analysis	0.95	0.04
Strongly enthusiastic based on an RR as targeted in the TTM2 study [17] with a SD similar to the TTM study [40]	0.86	0.07
Moderately enthusiastic based on an RR similar to Lascarrou et al. [41] with a SD similar to the TTM study [40]	0.98	0.07
Moderately sceptic based on an RR = 1 with a SD similar to the TTM study [40]	1	0.07
Strongly sceptic based on an RR = 1 with a SD half that of the TTM study [40]	1	0.035
Unfavourable neurological outcome		
Minimally informative	Cauchy distribution with location = 1	NA
Informed based on frequentist random effects meta-analysis	0.94	0.04
Strongly enthusiastic based on an RR as targeted in the TTM2 study [17] with a SD similar to the TTM study [40]	0.86	0.06
Moderately enthusiastic based on an RR similar to Lascarrou et al. [41] with a SD similar to the TTM study [40]	0.95	0.06
Moderately sceptic based on an RR = 1 with a SD similar to the TTM study [40]	1	0.06
Strongly sceptic based on an RR = 1 with a SD half that of the TTM study [40]	1	0.03

Based on 14,886 meta-analyses [26]:	Mean	Standard deviation
Between-study heterogeneity (*τ*^2^)		
Outcome: death	0.02	0.51
Outcome: unfavourable neurological recovery	0.02	1.23
Publication bias^a^		
Two-sided *p* value < 0.05	100% publication rate	
Two-sided *p* value ≥ 0.05 < 0.10	66% publication rate	
Two-sided *p* value ≥ 0.10	33% publication rate	

^a^Selection models use weighted distributions to account for the proportion of studies that are missing because they yielded non-significant results

All analyses were performed using RStudio (version 1.3.1093) [35] and JASP (version 0.14.1) [36] including the meta, RoBMA, metaBMA, MCMCpack packages.

## Results

**Fig. 1:**
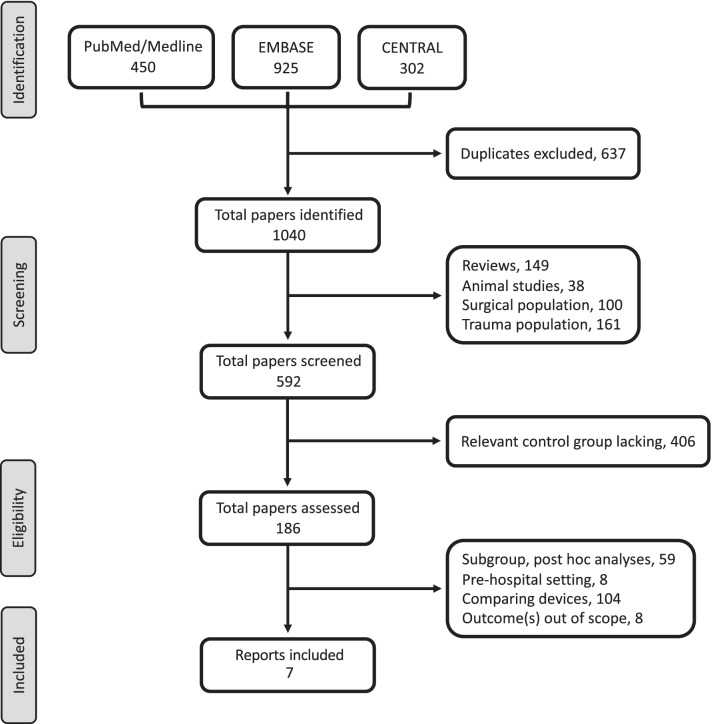
PRISMA flow diagram for original search (The expanded search PRISMA data are reported at bottom of in Additional File 1: Table S4)

The original and expanded search strategies generated 1040 and 4201 unique publications, respectively, of which 186 and 80 were assessed for full-text eligibility with the same 7 trials that recruited 3792 patients included in the final analyses (Fig. 1; Additional File 1: Table S4 bottom) [17, 37–42] of which 1898 received TTM. One study [42] reported neurological function at hospital discharge. A temperature of 32–34 °C was used in the intervention groups (TTM_32–34_). Patients in the control groups lacked explicit temperature definitions of normothermia in four studies [37–39, 42] while TTM at 36–37.5 °C and avoiding fever was applied in three studies [17, 40, 41] (TTM_≥36_). The majority of patients were OHCA with IHCA only included in two studies [37, 41]. The characteristics of included studies are reported in Table 2.

**Table 2:** Included randomised controlled studies on targeted temperature management after cardiac arrest

Study	Study setting	Years	Inclusion criteria	Number of patients	Intervention group	Control group	Primary outcome	Intervention group		Control group	
								Deaths/total	Unfavourable neurological outcome/total	Deaths/total	Unfavourable neurological outcome/total
Bernard [42]	Single centre in Melbourne, Australia	1996–1999	OHCA, initial rhythm VF, age > 18 (men) and > 50 (women)		TTM at 33 °C for 18 h	No TTM, unclear about fever treatment	Proportion of CPC 1–2 at hospital discharge	22/43	22/43	23/34	25/34
HACA [37]	Multiple centres in Europe	1996–2001	OHCA and some IHCA, shockable rhythm, witnessed, age 18–75	275	TTM at 32–34 °C, for 24 h	No TTM, unclear about fever treatment	Proportion of CPC 1–2 at six months	56/137	61/136	76/138	83/137
Laurent [38]	Single centre in Paris, France	2000–2002	OHCA only, all rhythms, age 18–75	42	TTM at 32–33 °C for 16 h	No TTM, unclear about fever treatment	Survival until six months	15/22	15/22	11/20	11/20
Hachimi-Idrissi [39]	Single centre in Belgium	1999–2002	OHCA only, non-shockable rhythm, age > 18 years	28	TTM at 33 °C for 24 h	No TTM, unclear about fever treatment	Level of s100b	6/14	8/ 14	8/14	11/14
Nielsen [40]	Multiple centres in Europe and Australia	2010–2103	OHCA, all rhythms, age > 18	950	TTM at 33 °C for28h	TTM at 36 °C for 28 h	All-cause mortality until six months	226/473	251/469	220/466	242/464
Lascarrou [41]	Multiple centres in France	2014–2018	OHCA or IHCA, non-shockable rhythm, age > 18	584	TTM at 33 °C for 24 h	TTM at 36.5–37.5 °C	Proportion of CPC 1–2 at 90 days	231/284	255/284	247/297	280/297
Dankiewicz [17]	Multiple centres in Europe, Australia, New Zealand and USA	2017–2020	OHCA, all rhythms, age > 18	1900	TTM at 33 °C for 24 h	TTM, if > 37.8 °C then 37.5 °C	All-cause mortality until six months	465/925	488/881	446/925	479/866

*OHCA* out-of-hospital cardiac arrest, *VF* ventricular fibrillation, *TTM* targeted temperature management, *HACA* hypothermia after cardiac arrest, *IHCA* in-hospital cardiac arrest, *CPC* cerebral performance category

**Fig. 2:**
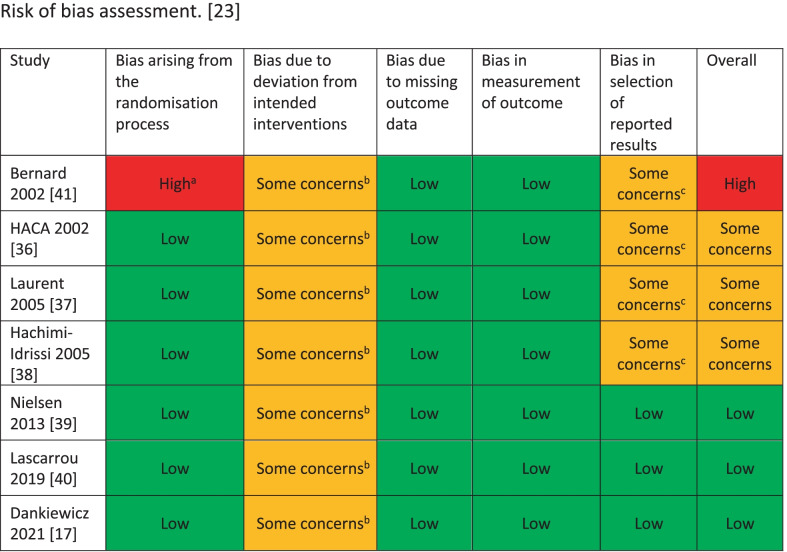
Risk of bias assessment for the included studies using the updated Cochrane RoB2 tool for randomised trials [23]. For each of the bias domains, the risk was classified as ‘low risk’, ‘some concerns’ or ‘high risk’. ^a^Randomisation by odd or even date of month; ^b^intervention not blinded to treating clinicians; ^c^no published pre-specified statistical analysis plan

Three studies were assessed as having low risk of bias, three studies with some concerns relating to the unblinding of the intervention and lack of pre-published protocols or statistical analysis plans and one with further high risk given its quasi-randomised design (Fig. 2). The certainty of evidence was graded as very low for survival and low for neurological outcome (Additional File 1: Table S5).

**Fig. 3:**
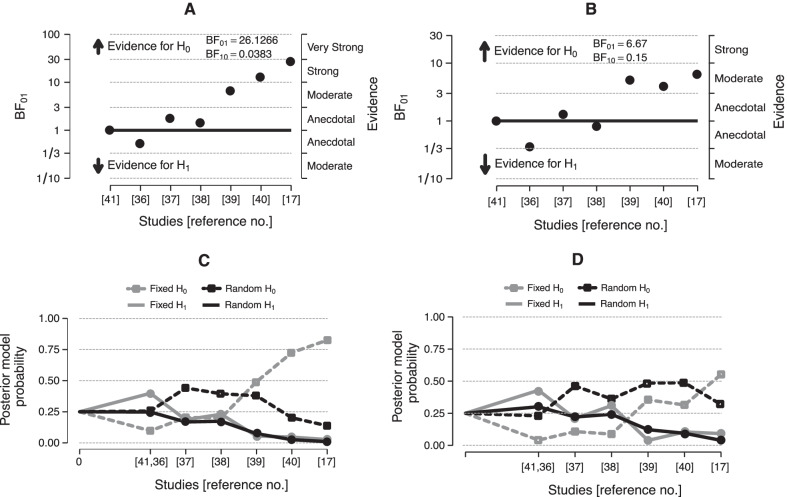
Diagram of the Bayes factor comparing the null hypothesis (H_0_, no difference between TTM_32–34_ and TTM_≥36_) and the alternative hypothesis (H_1_, TTM_32–34_ confers benefit compared with TTM_≥36_) with incremental evidence generated by the reviewed studies for death (**A**) and unfavourable neurological outcome (**B**). The posterior model probabilities for the H_0_ and H_1_ hypotheses in fixed and random effects models with incremental evidence are shown for death (**C**) and unfavourable neurological outcome (**D**)

The posterior likelihood favoured the null-hypothesis (H_0_) of no difference in deaths between TTM_32–34_ and TTM_≥36_ groups with BF_01_ = 26.1, i.e. the evidence for H_0_ is 26 times more likely than for H_1_ (Fig. 3A). The final posterior probability of the null hypothesis with sequential addition of studies was 82% in a fixed effects model and 14% in a random effects model (Fig. 3C). Conversely, the posterior probability supporting the hypothesis of benefit by TTM_32–34_ was 3% in a fixed and 1% in a random effects model (Fig. 3C). The posterior likelihood favoured the null hypothesis of no difference in neurological outcome between TTM_32–34_ and TTM_≥36_ groups with BF_01_ = 6.67, i.e. the evidence for H_0_ is 7 times more likely than for H_1_ (Fig. 3B). Incremental study data generated a posterior probability for the null hypothesis of 53% and 28% in fixed and random models, respectively, with a posterior probability for the hypothesis of benefit by TTM_32–34_ at 7% in a fixed and 2% in a random model (Fig. 3D).

**Fig. 4:**
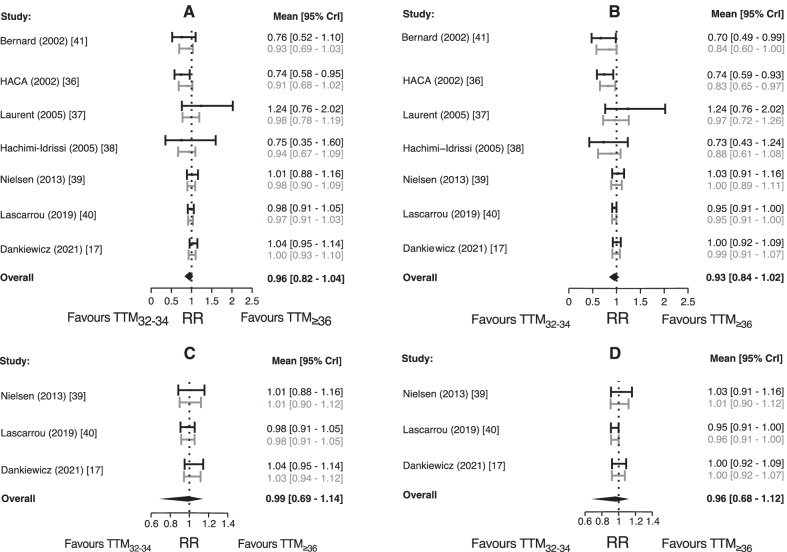
Hierarchical robust Bayesian model-averaged meta-analysis of the effect of TTM on deaths (left hand graphs) and unfavourable neurological outcome (right hand graphs) considering all studies (**A** and **B**) and studies with an explicit temperature definition of normothermia and avoidance of fever in the control group (**C** and **D**). Black bars and text refer to the observed study effect. Grey bars and text refer to the estimated study effect. The model-averaged effect (overall) in bold black text and black diamond. *RR* risk ratio; *95% CrI* 95% credible interval for the posterior probability distribution

The Bayesian meta-analysis of survival demonstrated a mean RR for death 0.96 (95% CrI 0.82–1.04) (Fig. 4A), with evidence for heterogeneity (*τ*), BF = 225, but without evidence of publication bias, BF = 1.24. The Bayesian meta-analysis of neurological recovery demonstrated a mean RR for unfavourable neurological outcome 0.93 (95% CrI 0.84–1.02) (Fig. 4B), again with evidence for heterogeneity (*τ*), BF = 308 but not for publication bias, BF = 1.11. These RRs remained unchanged in the sensitivity analyses using a range of effect size priors (Additional File 1: Table S6). In the sensitivity analyses excluding studies without explicit temperature definition of normothermia and avoidance of fever in the control group, the heterogeneity was substantially reduced (BF = 33 for survival and BF = 35 for neurological outcome). The RR for death changed to 0.99 (95% CrI 0.69–1.14) (Fig. 4C) and the RR for unfavourable neurological outcome to 0.96 (95% CrI 0.68–1.12) (Fig. 4D).

The posterior probabilities for treatment effects at different thresholds are given in Table 3 for death and in Table 4 for neurological outcome.

**Table 3:** Posterior probability (%) of treatment effect by specified threshold criteria for death for all studies (top) and only for studies with explicit temperature definition of normothermia and using fever avoidance in the control group (bottom)

No benefit RR ≥ 1	Any benefit RR < 1	ARR > 2%	ARR > 4%	ARR > 6%	ARR 7.5%	ARR > 10%
Death up to 180 days [17, 36–41]						
24	76	53	28	11	4	1
Death up to 180 days Explicit temperature definition of normothermia and using fever avoidance in control group [17, 39, 40]						
45	55	42	30	20	14	7

*RR* risk ratio, *ARR* absolute risk reduction

**Table 4:** Posterior probability (%) of treatment effect by specified threshold criteria for unfavourable neurological outcome censored at 90–180 days following cardiac arrest (top) or between hospital discharge to 180 days (middle)

No benefit RR ≥ 1	Any benefit RR < 1	ARR > 2%	ARR > 4%	ARR > 6%	ARR 7.5%	ARR > 10%
Unfavourable neurological outcome at 90–180 days [17, 36–40]						
17	83	67	47	28	17	6
Unfavourable neurological outcome, hospital discharge to 90–180 days [17, 36–41]						
12	88	78	63	45	32	14
Unfavourable neurological outcome, 90–180 days explicit temperature definition of normothermia and using fever avoidance in control group [17, 39, 40]						
37	63	50	36	25	17	9

The bottom row only includes studies with explicit temperature definition of normothermia and using fever avoidance in the control group (bottom)

*RR* risk ratio, *ARR* absolute risk reduction

The posterior probability of no benefit conferred (RR ≥ 1) by TTM_32–34_ compared to TTM_≥36_ was 24% and 12% for death and unfavourable neurological outcome, respectively. Minor treatment effects of TTM_32–34_ at ARR of 2–4% had the greatest posterior probabilities (28–78%). Including short-term neurological outcome at hospital discharge increased the posterior probabilities of benefit by TTM_32–34_ and extended the range where chances of benefit outweighed no-benefit up to ARR > 10%. The probability for no benefit of TTM_32–34_ (RR ≥ 1) increased to 45% for death and to 37% for unfavourable neurological outcome with the probabilities for achieving an ARR 2% ≤ 50% and for ARR 4% less than the chance of no benefit when only studies with explicit temperature definition of normothermia and avoidance of fever in the control groups were considered. In patients with an initial shockable versus non-shockable rhythm, the RR for death was 0.91 (95% CrI 0.66–1.06) versus 1.00 (95% CrI 0.93–1.07) (Additional File 1: Fig. S2) and for unfavourable neurological outcome 0.87 (95% CrI 0.60–1.04) versus 0.89 (95% CrI 0.57–1.09) (Additional File 1: Fig. S3).

## Discussion

This systematic review of randomised controlled trials of TTM in post-cardiac arrest care used Bayesian meta-analysis to assess posterior probabilities for a range of effect sizes by hypothermia (TTM_32–34_) compared with normothermia (TTM_≥36_) with or without avoidance of fever on survival and neurological recovery. The likelihood for the hypothesis of no difference between TTM_32–34_ and TTM_≥36_ was 26 times that of benefit by TTM_32–34_ for death and 8 times for unfavourable neurological recovery. The posterior probability of a 7.5–10% absolute risk reduction by TTM_32–34_ as targeted in the included trials was < 20% and only the posterior probabilities for achieving 2–4% absolute risk reductions were greater than the chances of no benefit conferred. However, in sensitivity analyses excluding studies without explicit temperature definition of normothermia and avoidance of fever, the posterior probability for no benefit by TTM_32–34_ was similar to or greater than any benefit in survival while the risk–benefit balance was slightly supportive only for achieving a 2% absolute risk reduction for unfavourable neurological outcome.

A Bayesian meta-analysis was chosen for this review as it can incorporate external data such as results from other meta-analyses or clinicians’ views on effect sizes. It can also demonstrate the relationship between treatment risk and benefit [43] within a limited number of studies [44] as the observed treatment effect of one trial is informed by that of the other trials. The aggregate of the estimated intervention effect is thus less susceptible to trials with small or extreme results. The 95% credible interval of posterior probabilities allows for a range of treatment effects to be evaluated which might be attractive to clinicians considering individualised TTM in post-cardiac arrest patients influenced by idiosyncratic factors associated with presence or absence of any benefit.

The rejection or acceptance of a hypothesis model is common to scientific inquiry. This study evaluated the null hypothesis (H_0_) of no difference between patients treated with TTM at 32–34 °C compared to TTM ≥ 36 °C with or without treating fever versus the alternative hypothesis (H_1_) that hypothermic TTM confers a benefit. The Bayesian analysis illustrated the likelihood of the body of evidence considered in the rejection or acceptance of the TTM hypothesis, notwithstanding the very low to low certainty in the evidence. The incremental study data provided strong evidence (BF = 26) in support of H_0_ for death and substantial evidence (BF = 8) for H_0_ regarding neurological outcome. The posterior probabilities for the alternative hypothesis (H_1_) of benefit by TTM_32–34_ were < 10% in both the fixed effect, i.e. all studies share a common treatment effect, and the random effect, i.e. studies share a distribution of treatment effects, models. There is thus an absence of evidence to support TTM_32–34_ for survival given the 1–3% posterior probability. Furthermore, there is plausible absence of evidence for neurological outcome based on the 2–7% posterior probability. It must be noted that the posterior probability in the random effects model for the hypothesis of equipoise (H_0_) was around 25% for both outcomes in the random effects model that is less convincing and suggests further trials are needed comparing TTM_≥36_ with other temperatures.

Previous frequentist meta-analyses of TTM have reported an overall (considering all studies regardless of initial rhythm, TTM characteristics and times for outcome assessment) non-significant effect on survival and a variable but mostly favourable effect on neurological outcomes by TTM_32–34_ [6, 7, 9, 10, 45]. Reviews of TTM in post-cardiac arrest care using expanded inclusion criteria and including retrospective, observational cohort studies, while unsuitable to inform clinical practice, have reported a benefit of TTM_32–34_ [4, 46]. Two recent meta-analyses including the TTM2 trial concluded that various levels of hypothermic TTM may not improve survival or neurological outcome compared to normothermia [47, 48] while associated with higher incidence of arrhythmias [48]. The Bayesian meta-analysis used in this review allowed for extended observations. First, the posterior probability distribution for death and unfavourable neurological outcome beyond a risk ratio ≥ 1 indicated when TTM_32–34_ was at best futile and at worst could potentially be associated with harm. These probabilities (24% for death and 12% for unfavourable neurological outcome in all studies and 45% and 37%, respectively, in the sensitivity analysis) warrant careful consideration as TTM_32–34_ is not devoid of adverse effects, e.g. arrhythmias, prolonged mechanical ventilation and extended stay in ICU [17, 34]. Second, the posterior probabilities of benefit by RR < 1 were highest for an ARR of 2–4%. There was a 28–67% chance of achieving this result by TTM_32–34_ for death and unfavourable neurological outcome in all studies but this was reduced to 30–50% in the sensitivity analysis. Such magnitudes of treatment effects might not be discernible or convincing in the local context of providing care to comatose survivors of cardiac arrest but could still carry benefits on a hospital network or population level. An international survey of individuals involved in cardiac resuscitation reported a minimum clinically important difference of 2–6% for survival with good neurological outcome at hospital discharge across different cardiac arrest characteristics [33]. However, this Bayesian analysis demonstrated that at a level where 25 patients need to be treated to gain one survivor, the chance of benefit by TTM_32–34_ is almost on par with the absence of any benefit. This challenges the routine use of hypothermic TTM. In terms of neurological outcome at a similar risk reduction level the balance of probabilities was in favour of TTM_32–34_, but arguably still not forming a compelling case for routine hypothermic TTM. Third, treatment effects by TTM_32–34_ at an ARR of 7.5–10%, while typically targeted in the sample size calculations for the included RCTs, had very low chances (1–17%) of being achieved. Fourth, in sensitivity analyses for a range of effect size priors including strongly enthusiastic as well as sceptic beliefs on TTM, the RR and 95% CrI remained principally unchanged and support the overall RR estimate as robust. The posterior probabilities of benefit by TTM_32–34_ on survival and neurological recovery were however shifted towards benefit in patients with an initial shockable compared to non-shockable rhythm although this difference was substantially reduced if studies not using a specific temperature to define normothermia in the control group were removed.

Study heterogeneity in previous [4–10] and recent [47–50] systematic reviews and meta-analyses has been moderate to high [51] and largely related to studies where hypothermia was compared to no temperature control including a proportion of patients with febrile temperatures. The addition of the recent study by Dankiewicz et al. [17] to the cumulative evidence has reduced heterogeneity given its sample size and protocolised avoidance of fever, similar to the original study by Nielsen et al. [40]. Notably, approximately 40% of patients in the study by Dankiewicz et al. [17] required active temperature management to maintain normothermia. The Bayesian meta-analysis demonstrated an approximate ten-fold reduction in the posterior probability of heterogeneity by excluding studies without explicit temperature definition of normothermia and avoiding fever and justified the sensitivity analysis of the remaining studies. The evidence still supported remaining heterogeneity and differences in the design, conduct and reporting of studies as well as general post-cardiac arrest care are essential considerations in the translation of research results into practice. It seems that clinicians may have interpreted the frequentist dichotomy driven by a threshold *p* value as evidence of absence of a treatment effect in non-significant (*p* > 0.05) TTM studies. The Nielsen et al. trial that reported no significant difference between TTM at 33 and 36 °C [40] was followed by a decreased use of TTM with increased incidence of febrile temperatures in cardiac arrest patients admitted to ICU [52–54]. It would seem prudent to avoid a similar dismissal of TTM [55] after the most recent trial not at least considering that the posterior probabilities for any benefit by hypothermic TTM still remained greater than the risk of no benefit in the sensitivity analyses. The importance of fever prevention also warrants further investigation. Several aspects of TTM remain the subject of debate and ongoing clinical research, including patient selection (e.g. shockable vs. non-shockable rhythms), timing and speed of instituting TTM, the optimal target temperature, duration, mode of reducing body temperature and management of the rewarming phase. The RCTs to date have been underpowered to detect the most likely effect size estimates of 2–4% demonstrated in this meta-analysis. A future RCT aimed at this effect using frequentist statistical inference would require prohibitively large study populations with between 2500 and 9000 participants per arm of the trial. Adaptive platform trial designs that employ Bayesian statistical inference might prove more realistic. Such future adaptive trials could investigate multiple domains of TTM management and assign more enthusiastic priors to early initiation of TTM, shockable rhythms, neurological outcomes with a sceptic prior for hypothermic TTM and an informed prior for TTM_≥36_ compared to higher temperatures. Enrichment strategies could be used to identify patients for whom a reduced risk by 2–6% is meaningful.

### Strengths and limitations

This systematic review and meta-analysis is strengthened by incorporating the study by Dankiewicz et al. [17] that doubles the study population compared to the previous literature. The use of Bayesian statistical inference to evaluate TTM has not been reported before and allowed treatment effects to be evaluated on a continuous spectrum of probabilities. The informed prior was aligned with the results of recent frequentist systematic reviews and meta-analyses [47, 48] and may thus be considered to reflect best current evidence. Several important limitations should be noted. The analysis comprised studies conducted predominantly in European hospitals. Other synonyms/permutations in the search strings might be considered although no additional studies relevant to this analysis have been identified in other recent systematic reviews using other comprehensive search strategies [47–50]. The study by Dankiewicz et al. [17] contributed 50% of the patient cohort and is hence influential on the results. Other important patient-centred outcomes such as quality of life or cognitive function and outcomes beyond 180 days were not included. This review compared hypothermic TTM at 32–34 °C with TTM at ≥ 36 °C and thus variable effects within the hypothermic range, e.g. 31.5 versus 34 °C (CAPITAL-CHILL, NCT02011568) or 32 versus 34 °C [56] were not explored. The results are influenced by the variable quality of included studies and neither adjunct therapies, details of TTM management nor determination of futility could be consistently assessed. While data-driven priors were used in this study, other settings for priors may be used and could generate different posterior distributions of probabilities [43, 57, 58]. In settings where the baseline incidence of death or unfavourable neurological outcome is higher or lower, the thresholds in the distribution of posterior probabilities for the ARR illustrations would correspond to higher and lower chances, respectively, to achieve similar reductions in RR.

## Conclusions

This Bayesian meta-analysis of randomised controlled trials of target temperature management (TTM) for at least twelve hours in adult comatose survivors of cardiac arrest did not support the use of TTM at 32–34 °C as compared to ≥ 36 °C also including active control of fever, to reduce the risk of death and unfavourable neurological outcome at 90–180 days. Future studies would need to consider that the most probable effect size estimates of TTM are less than half of that targeted in trials to date.

## Supplementary Information

**Additional file 1. Table 1**. PRIMSA checklist.** Table 2**. ROBUST criteria.** Table 3**. Sample search string.** Table 4**. Settings of the data driven priors for the primary outcome mortality and secondary outcome unfavourable neurology at 90-180 days.** Table 5**. GRADE assessment of certainty of evidence.** Table 6**. Sensitivity analyses using different effect size priors for the Bayesian meta-analysis.** Figure 1**. Frequentist meta-analysis of death and unfavourable neurological oucome.** Figure 2**. Sensitivity analysis for survival comparing initial shockable vs. non-shockable rhythms.** Figure 3**. Sensitivity analysis for neurological outcome comparing initial shockable vs. non-shockable rhythms.

## Data Availability

The data are available upon reasonable request and published source data are already available in the public domain. No custom code was used and statistical packages are stated in Methods and available in the public domain.

## Abbreviations

95% CI: 95% Confidence interval
95% CrI: 95% Credible interval
ARR: Absolute risk reduction
GRADE: Grading of Recommendation Assessment, Development and Evaluation
RCT: Randomised controlled trial
RoB: Risk of bias
RR: Risk ratio
TTM: Target temperature management

## References

[1] Donnino MW, Andersen LW, Berg KM, Reynolds JC, Nolan JP, Morley PT, Lang E, Cocchi MN, Xanthos T, Callaway CW (2016). Temperature management after cardiac arrest: an advisory statement by the Advanced Life Support Task Force of the International Liaison Committee on Resuscitation and the American Heart Association Emergency Cardiovascular Care Committee and the Council on Cardiopulmonary, Critical Care, Perioperative and Resuscitation. Resuscitation.

[2] Panchal AR, Bartos JA, Cabanas JG, Donnino MW, Drennan IR, Hirsch KG, Kudenchuk PJ, Kurz MC, Lavonas EJ, Morley PT (2020). Part 3: adult basic and advanced life support: 2020 American Heart Association guidelines for cardiopulmonary resuscitation and emergency cardiovascular care. Circulation.

[3] Nolan JP, Sandroni C, Bottiger BW, Cariou A, Cronberg T, Friberg H, Genbrugge C, Haywood K, Lilja G, Moulaert VRM (2021). European Resuscitation Council and European Society of Intensive Care Medicine guidelines 2021: post-resuscitation care. Resuscitation.

[4] Barbarawi M, Alabdouh A, Barbarawi O, Lakshman H, Alkasasbeh M, Rizk F, Bachuwa G, Alkotob ML (2020). Targeted temperature management in cardiac arrest patients with an initial non-shockable rhythm: a systematic review and meta-analysis. Shock.

[5] Alqalyoobi S, Boctor N, Sarkeshik AA, Hoerger J, Klimberg N, Bartolome BG, Stewart SL, Albertson TE (2019). Therapeutic hypothermia and mortality in the intensive care unit: systematic review and meta-analysis. Crit Care Resusc.

[6] Arrich J, Holzer M, Havel C, Mullner M, Herkner H (2016). Hypothermia for neuroprotection in adults after cardiopulmonary resuscitation. Cochrane Database Syst Rev.

[7] Bhattacharjee S, Baidya DK, Maitra S (2016). Therapeutic hypothermia after cardiac arrest is not associated with favorable neurological outcome: a meta-analysis. J Clin Anesth.

[8] Hakim SM, Ammar MA, Reyad MS (2018). Effect of therapeutic hypothermia on survival and neurological outcome in adults suffering cardiac arrest: a systematic review and meta-analysis. Minerva Anestesiol.

[9] Kalra R, Arora G, Patel N, Doshi R, Berra L, Arora P, Bajaj NS (2018). Targeted temperature management after cardiac arrest: systematic review and meta-analyses. Anesth Analg.

[10] Mahmoud A, Elgendy IY, Bavry AA (2016). Use of targeted temperature management after out-of-hospital cardiac arrest: a meta-analysis of randomized controlled trials. Am J Med.

[11] Goligher EC, Tomlinson G, Hajage D, Wijeysundera DN, Fan E, Juni P, Brodie D, Slutsky AS, Combes A (2018). Extracorporeal membrane oxygenation for severe acute respiratory distress syndrome and posterior probability of mortality benefit in a post hoc bayesian analysis of a randomized clinical trial. JAMA.

[12] Ryan EG, Harrison EM, Pearse RM, Gates S (2019). Perioperative haemodynamic therapy for major gastrointestinal surgery: the effect of a Bayesian approach to interpreting the findings of a randomised controlled trial. BMJ Open.

[13] Zampieri FG, Damiani LP, Bakker J, Ospina-Tascon GA, Castro R, Cavalcanti AB, Hernandez G (2020). Effects of a resuscitation strategy targeting peripheral perfusion status versus serum lactate levels among patients with septic shock. A Bayesian reanalysis of the ANDROMEDA-SHOCK trial. Am J Respir Crit Care Med.

[14] Yarnell CJ, Abrams D, Baldwin MR, Brodie D, Fan E, Ferguson ND, Hua M, Madahar P, McAuley DF, Munshi L (2021). Clinical trials in critical care: Can a Bayesian approach enhance clinical and scientific decision making?. Lancet Respir Med.

[15] Jacobs AK, Kushner FG, Ettinger SM, Guyton RA, Anderson JL, Ohman EM, Albert NM, Antman EM, Arnett DK, Bertolet M (2013). ACCF/AHA clinical practice guideline methodology summit report: a report of the American College of Cardiology Foundation/American Heart Association Task Force on Practice Guidelines. J Am Coll Cardiol.

[16] Guidance for the use of Bayesian statistics in medical device clinical trials. https://www.fda.gov/regulatory-information/search-fda-guidance-documents/guidance-use-bayesian-statistics-medical-device-clinical-trials-pdf-version.

[17] Dankiewicz J, Cronberg T, Lilja G, Jakobsen JC, Levin H, Ullen S, Rylander C, Wise MP, Oddo M, Cariou A (2021). Hypothermia versus normothermia after out-of-hospital cardiac arrest. N Engl J Med.

[18] Dankiewicz J, Cronberg T, Lilja G, Jakobsen JC, Belohlavek J, Callaway C, Cariou A, Eastwood G, Erlinge D, Hovdenes J (2019). Targeted hypothermia versus targeted Normothermia after out-of-hospital cardiac arrest (TTM2): a randomized clinical trial-Rationale and design. Am Heart J.

[19] Jakobsen JC, Dankiewicz J, Lange T, Cronberg T, Lilja G, Levin H, Belohlavek J, Callaway C, Cariou A, Erlinge D (2020). Targeted hypothermia versus targeted normothermia after out-of-hospital cardiac arrest: a statistical analysis plan. Trials.

[20] Page MJ, McKenzie JE, Bossuyt PM, Boutron I, Hoffmann TC, Mulrow CD, Shamseer L, Tetzlaff JM, Akl EA, Brennan SE (2021). The PRISMA 2020 statement: an updated guideline for reporting systematic reviews. BMJ.

[21] Sung L, Hayden J, Greenberg ML, Koren G, Feldman BM, Tomlinson GA (2005). Seven items were identified for inclusion when reporting a Bayesian analysis of a clinical study. J Clin Epidemiol.

[22] Steinbusch CVM, van Heugten CM, Rasquin SMC, Verbunt JA, Moulaert VRM (2017). Cognitive impairments and subjective cognitive complaints after survival of cardiac arrest: a prospective longitudinal cohort study. Resuscitation.

[23] Perkins GD, Callaway CW, Haywood K, Neumar RW, Lilja G, Rowland MJ, Sawyer KN, Skrifvars MB, Nolan JP (2021). Brain injury after cardiac arrest. Lancet.

[24] Sterne JAC, Savovic J, Page MJ, Elbers RG, Blencowe NS, Boutron I, Cates CJ, Cheng HY, Corbett MS, Eldridge SM (2019). RoB 2: a revised tool for assessing risk of bias in randomised trials. BMJ.

[25] Guyatt GH, Oxman AD, Vist GE, Kunz R, Falck-Ytter Y, Alonso-Coello P, Schunemann HJ, Group GW (2008). GRADE: an emerging consensus on rating quality of evidence and strength of recommendations. BMJ.

[26] Heck DW, Gronau QF, Wagenmakers E-J. metaBMA: Bayesian model averaging for random and fixed effects meta-analysis. https://CRAN.R-project.org/package=metaBMA; 2019.

[27] Bartos F, Maier M. RoBMA: an R package for robust Bayesian meta-analyses. https://CRAN.R-project.org/package=RoBMA. 2020.

[28] Gronau QF, Heck DW, Berkhout SW, Haaf JM, Wagenmakers E. A primer on Bayesian model-averaged meta-analysis. PsyArXiv Preprints; 2020. 10.3758/s13428-023-02093-6 PMC10991068 37099263

[29] Zampieri FG, Casey JD, Shankar-Hari M, Harrell FE, Jr., Harhay MO. Using Bayesian methods to augment the interpretation of critical care trials. An overview of theory and example reanalysis of the alveolar recruitment for acute respiratory distress syndrome trial. Am J Respir Crit Care Med. 2021;203(5):543–552. 10.1164/rccm.202006-2381CP PMC7924582 33270526

[30] Turner RM, Jackson D, Wei Y, Thompson SG, Higgins JP (2015). Predictive distributions for between-study heterogeneity and simple methods for their application in Bayesian meta-analysis. Stat Med.

[31] Virani SS, Alonso A, Benjamin EJ, Bittencourt MS, Callaway CW, Carson AP, Chamberlain AM, Chang AR, Cheng S, Delling FN (2020). Heart disease and stroke statistics-2020 update: a report from the American Heart Association. Circulation.

[32] Grasner JT, Wnent J, Herlitz J, Perkins GD, Lefering R, Tjelmeland I, Koster RW, Masterson S, Rossell-Ortiz F, Maurer H (2020). Survival after out-of-hospital cardiac arrest in Europe—results of the EuReCa TWO study. Resuscitation.

[33] Nichol G, Brown SP, Perkins GD, Kim F, Sterz F, Broeckel Elrod JA, Mentzelopoulos S, Lyon R, Arabi Y, Castren M (2016). What change in outcomes after cardiac arrest is necessary to change practice? Results of an international survey. Resuscitation.

[34] Johnsson J, Wahlstrom J, Dankiewicz J, Annborn M, Agarwal S, Dupont A, Forsberg S, Friberg H, Hand R, Hirsch KG (2020). Functional outcomes associated with varying levels of targeted temperature management after out-of-hospital cardiac arrest—an INTCAR2 registry analysis. Resuscitation.

[35] RStudio Team: RStudio: integrated development environment for R; 2020.

[36] JASP Team: JASP; 2020.

[37] Hypothermia after Cardiac Arrest Study G. Mild therapeutic hypothermia to improve the neurologic outcome after cardiac arrest. N Engl J Med. 2002;346(8):549–56. 10.1056/NEJMoa012689 11856793

[38] Laurent I, Adrie C, Vinsonneau C, Cariou A, Chiche JD, Ohanessian A, Spaulding C, Carli P, Dhainaut JF, Monchi M (2005). High-volume hemofiltration after out-of-hospital cardiac arrest: a randomized study. J Am Coll Cardiol.

[39] Hachimi-Idrissi S, Zizi M, Nguyen DN, Schiettecate J, Ebinger G, Michotte Y, Huyghens L (2005). The evolution of serum astroglial S-100 beta protein in patients with cardiac arrest treated with mild hypothermia. Resuscitation.

[40] Nielsen N, Wetterslev J, Cronberg T, Erlinge D, Gasche Y, Hassager C, Horn J, Hovdenes J, Kjaergaard J, Kuiper M (2013). Targeted temperature management at 33 degrees C versus 36 degrees C after cardiac arrest. N Engl J Med.

[41] Lascarrou JB, Merdji H, Le Gouge A, Colin G, Grillet G, Girardie P, Coupez E, Dequin PF, Cariou A, Boulain T (2019). Targeted temperature management for cardiac arrest with nonshockable rhythm. N Engl J Med.

[42] Bernard SA, Gray TW, Buist MD, Jones BM, Silvester W, Gutteridge G, Smith K (2002). Treatment of comatose survivors of out-of-hospital cardiac arrest with induced hypothermia. N Engl J Med.

[43] Al Amer FM, Thompson CG, Lin L (2021). Bayesian methods for meta-analyses of binary outcomes: implementations, examples, and impact of priors. Int J Environ Res Public Health.

[44] Seide SE, Rover C, Friede T (2019). Likelihood-based random-effects meta-analysis with few studies: empirical and simulation studies. BMC Med Res Methodol.

[45] Rout A, Singh S, Sarkar S, Munawar I, Garg A, D'Adamo CR, Tantry US, Dharmadhikari A, Gurbel PA (2020). Meta-analysis of the usefulness of therapeutic hypothermia after cardiac arrest. Am J Cardiol.

[46] Schenone AL, Cohen A, Patarroyo G, Harper L, Wang X, Shishehbor MH, Menon V, Duggal A (2016). Therapeutic hypothermia after cardiac arrest: a systematic review/meta-analysis exploring the impact of expanded criteria and targeted temperature. Resuscitation.

[47] Granfeldt A, Holmberg MJ, Nolan JP, Soar J, Andersen LW (2021). International Liaison Committee on Resuscitation Advanced Life Support Task F: targeted temperature management in adult cardiac arrest: systematic review and meta-analysis. Resuscitation.

[48] Fernando SM, Di Santo P, Sadeghirad B, Lascarrou JB, Rochwerg B, Mathew R, Sekhon MS, Munshi L, Fan E, Brodie D (2021). Targeted temperature management following out-of-hospital cardiac arrest: a systematic review and network meta-analysis of temperature targets. Intensive Care Med.

[49] Elbadawi A, Sedhom R, Baig B, Mahana I, Thakker R, Gad M, Eid M, Nair A, Kayani W, Denktas A (2021). Targeted hypothermia vs targeted normothermia in survivors of cardiac arrest: a systematic review and meta-analysis of randomized trials. Am J Med.

[50] Sanfilippo F, La Via L, Lanzafame B, Dezio V, Busalacchi D, Messina A, Ristagno G, Pelosi P, Astuto M (2021). Targeted temperature management after cardiac arrest: a systematic review and meta-analysis with trial sequential analysis. J Clin Med.

[51] Higgins JP, Thompson SG, Deeks JJ, Altman DG (2003). Measuring inconsistency in meta-analyses. BMJ.

[52] Salter R, Bailey M, Bellomo R, Eastwood G, Goodwin A, Nielsen N, Pilcher D, Nichol A, Saxena M, Shehabi Y (2018). Changes in temperature management of cardiac arrest patients following publication of the target temperature management trial. Crit Care Med.

[53] Garfield B, Abdoolraheem MY, Dixon A, Aswani A, Paul R, Sherren P, Glover G (2020). Temporal changes in targeted temperature management for out-of-hospital cardiac arrest-examining the effect of the targeted temperature management trial: a retrospective cohort study. Ther Hypothermia Temp Manag.

[54] Nolan JP, Orzechowska I, Harrison DA, Soar J, Perkins GD, Shankar-Hari M (2021). Changes in temperature management and outcome after out-of-hospital cardiac arrest in United Kingdom intensive care units following publication of the targeted temperature management trial. Resuscitation.

[55] Morrison LJ, Thoma B (2021). Translating targeted temperature management trials into postarrest care. N Engl J Med.

[56] Lopez-de-Sa E, Juarez M, Armada E, Sanchez-Salado JC, Sanchez PL, Loma-Osorio P, Sionis A, Monedero MC, Martinez-Selles M, Martin-Benitez JC (2018). A multicentre randomized pilot trial on the effectiveness of different levels of cooling in comatose survivors of out-of-hospital cardiac arrest: the FROST-I trial. Intensive Care Med.

[57] Wijeysundera DN, Austin PC, Hux JE, Beattie WS, Laupacis A (2009). Bayesian statistical inference enhances the interpretation of contemporary randomized controlled trials. J Clin Epidemiol.

[58] Johnson SR, Tomlinson GA, Hawker GA, Granton JT, Feldman BM (2010). Methods to elicit beliefs for Bayesian priors: a systematic review. J Clin Epidemiol.

