# Supplementary material for: Target temperature management following cardiac arrest: a systematic review and Bayesian meta-analysis
Source: Crit Care. 2022 Mar 12;26:58. doi: 10.1186/s13054-022-03935-z (PMC8917746; doi:10.1186/s13054-022-03935-z)

ADDITIONAL FILE
Target temperature management following cardiac arrest: a systematic review and Bayesian meta-analysis

Table 1. PRISMA checklist [20]

Section and Topic	Item #	Checklist item	Location where item is reported
**TITLE**			
Title	1	Identify the report as a systematic review.	Page 1, lines 1-2
**ABSTRACT**			
Abstract	2	See the PRISMA 2020 for Abstracts checklist.	Lines 24-53, as per Crit Care
**INTRODUCTION**			
Rationale	3	Describe the rationale for the review in the context of existing knowledge.	Lines 60-71
Objectives	4	Provide an explicit statement of the objective(s) or question(s) the review addresses.	Lines 77-85
**METHODS**			
Eligibility criteria	5	Specify the inclusion and exclusion criteria for the review and how studies were grouped for the syntheses.	Lines 92-103
Information sources	6	Specify all databases, registers, websites, organisations, reference lists and other sources searched or consulted to identify studies. Specify the date when each source was last searched or consulted.	Lines 105-112
Search strategy	7	Present the full search strategies for all databases, registers and websites, including any filters and limits used.	Additional file, Table 3 and PROSPERO registration
Selection process	8	Specify the methods used to decide whether a study met the inclusion criteria of the review, including how many reviewers screened each record and each report retrieved, whether they worked independently, and if applicable, details of automation tools used in the process.	Lines 127-129 and 120-121
Data collection process	9	Specify the methods used to collect data from reports, including how many reviewers collected data from each report, whether they worked independently, any processes for obtaining or confirming data from study investigators, and if applicable, details of automation tools used in the process.	Lines 114-120
Data items	10a	List and define all outcomes for which data were sought. Specify whether all results that were compatible with each outcome domain in each study were sought (e.g. for all measures, time points, analyses), and if not, the methods used to decide which results to collect.	Lines 118-120
	10b	List and define all other variables for which data were sought (e.g. participant and intervention characteristics, funding sources).	NA

Section and Topic	Item #	Checklist item	Location where item is reported
		Describe any assumptions made about any missing or unclear information.	
Study risk of bias assessment	11	Specify the methods used to assess risk of bias in the included studies, including details of the tool(s) used, how many reviewers assessed each study and whether they worked independently, and if applicable, details of automation tools used in the process.	Lines 124-127
Effect measures	12	Specify for each outcome the effect measure(s) (e.g. risk ratio, mean difference) used in the synthesis or presentation of results.	Lines 132-133
Synthesis methods	13a	Describe the processes used to decide which studies were eligible for each synthesis (e.g. tabulating the study intervention characteristics and comparing against the planned groups for each synthesis (item #5)).	As per #5 and #11
	13b	Describe any methods required to prepare the data for presentation or synthesis, such as handling of missing summary statistics, or data conversions.	Lines 132-133
	13c	Describe any methods used to tabulate or visually display results of individual studies and syntheses.	Table 1
	13d	Describe any methods used to synthesize results and provide a rationale for the choice(s). If meta-analysis was performed, describe the model(s), method(s) to identify the presence and extent of statistical heterogeneity, and software package(s) used.	Lines 133-179, Additional File, Table 4
	13e	Describe any methods used to explore possible causes of heterogeneity among study results (e.g. subgroup analysis, meta-regression).	Lines 140-143, 155-156, Additional File, Table 4
	13f	Describe any sensitivity analyses conducted to assess robustness of the synthesized results.	Lines 174-178, Additional File, Table 6
Reporting bias assessment	14	Describe any methods used to assess risk of bias due to missing results in a synthesis (arising from reporting biases).	As per RoB2
Certainty assessment	15	Describe any methods used to assess certainty (or confidence) in the body of evidence for an outcome.	Lines 157-159
**RESULTS**			
Study selection	16a	Describe the results of the search and selection process, from the number of records identified in the search to the number of studies included in the review, ideally using a flow diagram.	Figure 1
	16b	Cite studies that might appear to meet the inclusion criteria, but which were excluded, and explain why they were excluded.	Figure 1
Study characteristics	17	Cite each included study and present its characteristics.	Table 1
Risk of bias in studies	18	Present assessments of risk of bias for each included study.	Figure 2

Section and Topic	Item #	Checklist item	Location where item is reported
Results of individual studies	19	For all outcomes, present, for each study: (a) summary statistics for each group (where appropriate) and (b) an effect estimate and its precision (e.g. confidence/credible interval), ideally using structured tables or plots.	Figure 3, Additional File, Figure 2
Results of syntheses	20a	For each synthesis, briefly summarise the characteristics and risk of bias among contributing studies.	Lines 195-206
	20b	Present results of all statistical syntheses conducted. If meta-analysis was done, present for each the summary estimate and its precision (e.g. confidence/credible interval) and measures of statistical heterogeneity. If comparing groups, describe the direction of the effect.	Figure 3, Additional File, Figures 3 and 6
	20c	Present results of all investigations of possible causes of heterogeneity among study results.	Lines 211-214
	20d	Present results of all sensitivity analyses conducted to assess the robustness of the synthesized results.	Figure 3, Additional File, Figures 9 and 10, Table 6
Reporting biases	21	Present assessments of risk of bias due to missing results (arising from reporting biases) for each synthesis assessed.	Figure 2
Certainty of evidence	22	Present assessments of certainty (or confidence) in the body of evidence for each outcome assessed.	Additional File, Table 5
**DISCUSSION**			
Discussion	23a	Provide a general interpretation of the results in the context of other evidence.	Lines 225-237
	23b	Discuss any limitations of the evidence included in the review.	Lines 348-361
	23c	Discuss any limitations of the review processes used.	Lines 348-361
	23d	Discuss implications of the results for practice, policy, and future research.	Lines 326-339
**OTHER INFORMATION**			
Registration and protocol	24a	Provide registration information for the review, including register name and registration number, or state that the review was not registered.	Line 89
	24b	Indicate where the review protocol can be accessed, or state that a protocol was not prepared.	Line 89
	24c	Describe and explain any amendments to information provided at registration or in the protocol.	NA
Support	25	Describe sources of financial or non-financial support for the review, and the role of the funders or sponsors in the review.	Lines 397-

Section and Topic	Item #	Checklist item	Location where item is reported
			398
Competing interests	26	Declare any competing interests of review authors.	Lines 393-395
Availability of data, code and other materials	27	Report which of the following are publicly available and where they can be found: template data collection forms; data extracted from included studies; data used for all analyses; analytic code; any other materials used in the review.	Lines 388-391

Page MJ, McKenzie JE, Bossuyt PM, Boutron I, Hoffmann TC, Mulrow CD, et al. The PRISMA 2020 statement: an updated guideline for reporting systematic reviews. BMJ 2021;372:n71. doi: 10.1136/bmj.n71

Table 2. ROBUST criteria [21]

ITEM		LOCATION
**Prior distribution**	Specified	Lines 161-168, lines 183-197
	Justified	Lines 161-168, lines 1830197
	Sensitivity analysis	Lines 154-157
**Analysis**	Statistical model	Lines 138-154, lines 198-199
	Analytical technique	Line 138, lines 158-161
**Results**	Central tendency	Lines 145-147
	Standard deviation or credible interval	Lines 145-147

Sung L, Hayden J, Greenberg ML, Koren G, Feldman BM, Tomlinson GA. Seven items were identified for inclusion when reporting a Bayesian analysis of a clinical study. *J Clin Epidemiol.* 2005;58(3):261-268.

Table 3. Original search strategy

**PubMed/MEDLINE (ALL fields)**
#1 cardiac arrest [MeSH Major Topic]
#2 (targeted temperature management [MeSH Terms] OR induced hypothermia [MeSH Terms])
#3 trial [All fields]
#4 animals OR models, animals [All fields]
(#1 AND #2 AND #3) NOT #4

**EMBASE (Explode all terms)**	
#1	cardiac arrest.mp or heart arrest/
#2	limit 1 to human
#3	target* temperature management.mp or induced hypothermia/ or temperature/
#4	limit 3 to human
#5	clinical trial/ or controlled study/ or randomized controlled trial/ or controlled clinical trial/
#6	2 and 4 and 5

**CENTRAL (Cochrane Library)**	
#1	cardiac arrest
#2	MeSH descriptor: [Heart Arrest] explode all trees
#3	clinical trial
#4	MeSH descriptor: [Clinical Trial] explode all trees
#5	randomized clinical trial
#6	MeSH descriptor: [Randomized Controlled Trial] explode all trees
#7	targeted temperature management
#8	MeSH descriptor: [Hypothermia, Induced] explode all trees
#9	#1 OR #2
#10	#3 OR #4 OR #5 OR #6
#11	#7 OR #8
#12	#9 AND #10 AND #11

Table 4. Expanded search strategy.

PubMed

#1	(cardiac arrest[MeSH Terms]) OR (ventricular tachycardia[MeSH Terms]) OR (ventricular fibrillation[MeSH Terms]) OR (ventricular flutter[MeSH Terms]) OR (pulseless electrical activity[MeSH Terms]) OR (asystole[MeSH Terms]) OR (heart arrest[MeSH Terms]) OR (cardiopulmonary resuscitation[MeSH Terms]) OR (heart massage[MeSH Terms]) OR (return of spontaneous circulation[MeSH Terms]) OR (advanced cardiac life support[MeSH Terms]) OR (out of hospital cardiac arrest[MeSH Terms]) OR (in hospital cardiac arrest[MeSH Terms]) OR (OHCA[MeSH Terms]) OR (IHCA[MeSH Terms]) OR (ACLS[MeSH Terms]) OR (ROSC[MeSH Terms])	**88808**
#2	(cardiac arrest[Text Word]) OR (ventricular tachycardia[Text Word]) OR (ventricular fibrillation[Text Word]) OR (ventricular flutter[Text Word]) OR (pulseless electrical activity[Text Word]) OR (asystole[Text Word]) OR (heart arrest[Text Word]) OR (cardiopulmonary resuscitation[Text Word]) OR (heart massage[Text Word]) OR (return of spontaneous circulation[Text Word]) OR (advanced cardiac life support[Text Word]) OR (out of hospital cardiac arrest[Text Word]) OR (in hospital cardiac arrest[Text Word])) OR (OHCA[Text Word]) OR (IHCA[Text Word]) OR (ACLS[Text Word]) OR (ROSC[Text Word])	**114243**
#3	#1 OR #2	**131925**
#4	((temperature[Text Word]) OR (fever[Text Word])) AND ((target[Text Word] OR management[Text Word] OR control[Text Word])) OR (cool*[Text Word]) OR (hypothermi*[Text Word]) OR (therapeutic hypothermia[Text Word]) OR (TTM[Text Word]) OR (induced hypothermia[MeSH Terms]) OR (therapeutic hypothermia[MeSH Terms])	**288188**
#5	#3 AND #4	**10147**
#6	(randomized controlled trial[Publication Type]) OR (controlled clinical trial[Publication Type]) OR (randomized[Title/Abstract]) OR (randomly[Title/Abstract]))OR (trial[Title/Abstract]) OR (groups[Title/Abstract]) OR (placebo[Title/Abstract]) NOT ((animal*[Text Word] AND model*[Text Word]) OR animal*[Text Word])	**2766494**
#7	#5 AND #6	**1419**

EMBASE

1	(cardiac arrest or ventricular tachycardia or ventricular fibrillation or ventricular flutter or pulseless electrical activity or asystole or heart arrest or cardiopulmonary resuscitation or heart massage or return of spontaneous circulation or advanced cardiac life support or out of hospital cardiac arrest or in hospital cardiac arrest).mp. [mp=title, abstract, heading word, drug trade name, original title, device	**159371**

	manufacturer, drug manufacturer, device trade name, keyword heading word, floating subheading word, candidate term word]	
2	limit 1 to human	**132334**
3	(((temperature or fever) and (target or management or control)) or cool* or hypothermi* or therapeutic hypothermia or TTM or induced hypothermia).mp. [mp=title, abstract, heading word, drug trade name, original title, device manufacturer, drug manufacturer, device trade name, keyword heading word, floating subheading word, candidate term word]	**373545**
4	limit 3 to human	**195319**
5	(randomized controlled trial or controlled trial or randomized or randomly or trial or groups).mp. [mp=title, abstract, heading word, drug trade name, original title, device manufacturer, drug manufacturer, device trade name, keyword heading word, floating subheading word, candidate term word]	**5442035**
6	limit 5 to human	**4261503**
7	2 and 4 and 6	**3148**

Cochrane

#1	cardiac arrest or ventricular tachycardia or ventricular fibrillation or ventricular flutter or pulseless electrical activity or asystole or heart arrest or cardiopulmonary resuscitation or heart massage or return of spontaneous circulation or advanced cardiac life support or out of hospital cardiac arrest or in hospital cardiac arrest	**13615**
#2	((temperature or fever) and (target or management or control)) or cool* or hypothermi* or therapeutic hypothermia or TTM or induced hypothermia	**27378**
#3	randomized controlled trial or controlled trial or randomized or randomly or trial or groups	**1557611**
#4	#1 AND #2 AND #3 1514	**1514**

COVIDENCE (www.covidence.org) PRISMA data:

6081 references imported for screening as 6081 studies
- 1880 duplicates removed

4201 studies screened against title and abstract
- 4121 studies excluded

80 studies assessed for full-text eligibility
- 73 studies excluded
  - 35 Wrong study design
  - 19 Review
  - 12 Wrong comparator
  - 3 Wrong outcomes
  - 2 Wrong setting
  - 1 Wrong intervention
  - 1 Wrong patient population

7 studies included

Table 5. GRADE assessment of certainty of evidence [24]

Certainty assessment							No. of patients			
Number of studies	Design	Risk of bias	Inconsistency	Indirectness	Imprecision	Other	TTM	No TTM	Certainty	Importance
Death										
7	RCT	Not serious[a]	Not serious[b]	Not serious[d]	Very serious[e]	Older studies - larger effect on mortality[f]	1898	1894	⊕◯◯◯ VERY LOW	Critical
Unfavorable neurological outcome										
7	RCT	Not serious[a]	Not serious[b,c]	Not serious[d]	Serious[e]	None	1806	1798	⊕⊕◯◯ LOW	Critical

[a] Studies were not blinded but all outcomes were determined by blinded personnel. Three studies had strict protocols for prognostication which is likely to decrease the risk of bias
[b] All studies have shown either a positive or neutral effect, significant harm from TTM appears unlikely.
[c] Slightly inconsistent across sub-groups of OHCA and IHCA (Much larger effect size in the IHCA cohort in one RCT)
[d] Long-term endpoints of major importance for patients
[e] Studies with smaller sample size report larger effect
[f] Older studied showed larger effect
RCT=randomized controlled trial; TTM=target temperature managemen

Table 6. Sensitivity analyses using different effect size priors for the Bayesian meta-analysis. The risk ratio is reported as mean with its lower and upper 95% credibility interval (CrI).

Risk ratio for death			
**Prior description**	**Mean**	**Lower 95%CrI**	**Upper 95%CrI**
Minimally informative	0.96	0.82	1.04
Informed based on frequentist random effects meta-analysis	0.96	0.90	1.01
Strongly enthusiastic based on an RR as targeted in the TTM2 study [17] with a SD similar to the TTM study [35]	0.93	0.82	1.01
Moderately enthusiastic based on an RR similar to Lascarrou et al [41] with a SD similar to the TTM study [40]	0.95	0.84	1.02
Moderately sceptic based on an RR=1 with a SD similar to the TTM study [40]	0.98	0.90	1.04
Strongly sceptic based on an RR=1 with a SD half that of the TTM study [40]	0.99	0.94	1.03
**Risk ratio for unfavourable neurological outcome**			
**Prior description**	**Mean**	**Lower 95%CrI**	**Upper 95%CrI**
Minimally informative	0.93	0.84	1.02
Informed based on frequentist random effects meta-analysis	0.95	0.88	1.01
Strongly enthusiastic based on an RR as targeted in the TTM2 study [17] with a SD similar to the TTM study [40]	0.90	0.79	0.98
Moderately enthusiastic based on an RR similar to Lascarrou et al [41] with a SD similar to the TTM study [40]	0.93	0.84	1.02
Moderately sceptic based on an RR=1 with a SD similar to the TTM study [40]	0.95	0.87	1.05
Strongly sceptic based on an RR=1 with a SD half that of the TTM study [40]	0.97	0.88	1.04

Figure 1. Frequentist meta-analyses of survival (top) and neurological outcome (bottom).

A random effects model was used to generate the risk ratio comparing targeted temperature management (TTM) at 32-34˚C to ≥36˚C. The overall effect size, risk ratio (RR), is represented by the mean and 95% confidence interval.

Study (Year)	Hypothermia (n/N)(%)	Control (n/N)(%)	RR	95%–CI
**All trials**				
Bernard (2002) [42]	22/43 (51%)	23/34 (68%)	0.76	[0.47 - 1.05]
HACA (2002) [37]	56/137 (41%)	76/138 (55%)	0.74	[0.58 - 0.95]
Laurent (2005) [38]	15/22 (68%)	11/20 (55%)	1.24	[0.76 - 2.02]
Hachimi–Idrissi (2005) [39]	6/14 (43%)	8/14 (57%)	0.75	[0.35 - 1.60]
Nielsen (2013) [40]	226/473 (48%)	220/466 (47%)	1.01	[0.88 - 1.16]
Lascarrou (2019) [41]	231/284 (81%)	247/297 (83%)	0.98	[0.91 - 1.05]
Dankiewicz (2021) [17]	465/925 (50%)	446/925 (48%)	1.04	[0.95 - 1.14]
**Random effects model**			**0.95**	**[0.87 - 1.04]**

Heterogeneity: $I^2 = 47\%$, $\tau^2 = 0.0050$, $p = 0.12$

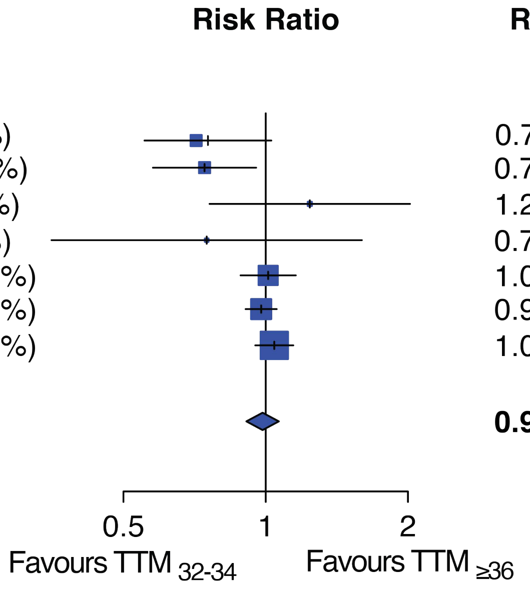

Study (Year)	Hypothermia (n/N)(%)	Control (n/N)(%)	RR	95%–CI
**All trials**				
Bernard (2002) [42]	22/43 (51%)	25/34 (74%)	0.80	[0.51 - 1.25]
HACA (2002) [37]	61/137 (45%)	83/138 (60%)	0.74	[0.59 - 0.93]
Laurent (2005) [38]	15/22 (68%)	11/20 (55%)	1.24	[0.76 - 2.02]
Hachimi–Idrissi (2005) [39]	8/14 (57%)	11/14 (79%)	0.73	[0.43 - 1.24]
Nielsen (2013) [40]	251/469 (54%)	242/464 (52%)	1.02	[0.88 - 1.16]
Lascarrou (2019) [41]	255/284 (90%)	280/297 (94%)	0.95	[0.91 - 1.00]
Dankiewicz (2021) [17]	488/881 (55%)	479/866 (55%)	1.00	[0.92 - 1.09]
**Random effects model**			**0.94**	**[0.87 - 1.02]**

Heterogeneity: $I^2 = 46\%$, $\tau^2 = 0.0033$, $p = 0.086$

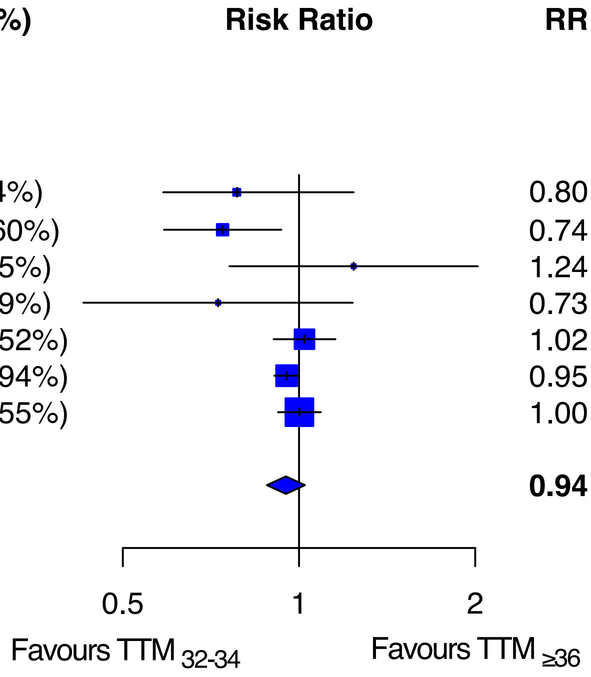

Figure 2. Sensitivity analysis: survival and shockable rhythm

Observed and estimated effects sizes on survival 90-180 days in patients with initial shockable (left graph) and non-shockable (right graph) rhythms. The observed effect sizes are displayed in black with the estimated effect size, i.e. the random effect estimate, displayed in grey. The overall effect size, risk ratio (RR), is represented by the mean and its 95% credible interval (95%CrI).

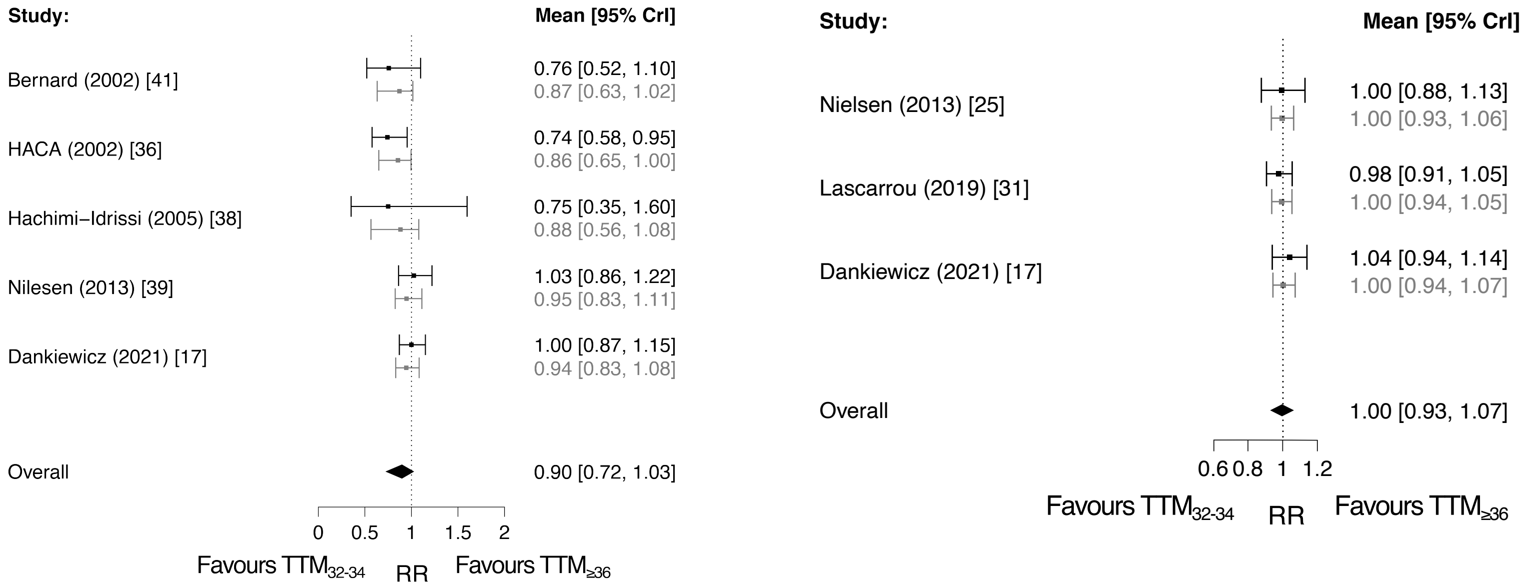

Figure 3. Sensitivity analysis: neurological outcome and shockable rhythm

Observed and estimated effects sizes on favourable neurological outcome 90-180 days in patients with initial shockable (left graph) and non-shockable (right graph) rhythms. The observed effect sizes are displayed in black with the estimated effect size, i.e. the random effect estimate, displayed in grey. The overall effect size, risk ratio (RR), is represented by the mean and its 95% credible interval (95%CrI).

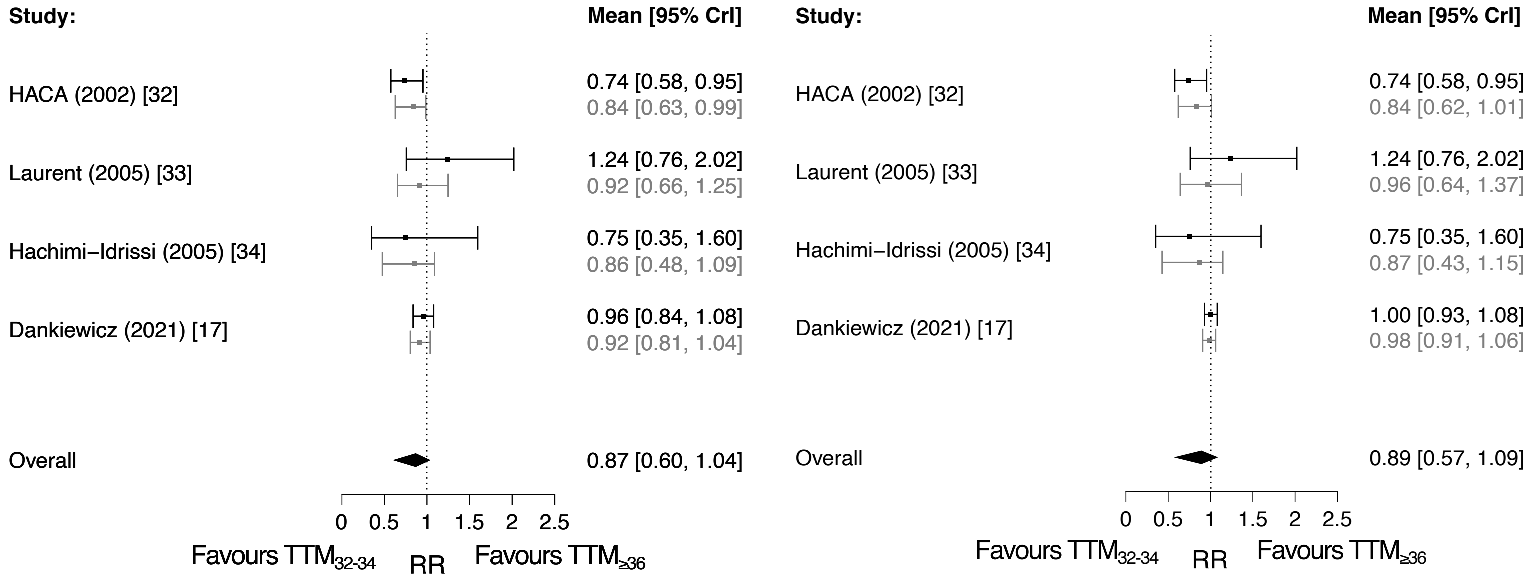

Supplement: Supplementary file 1 — Additional file 1. Table 1. PRIMSA checklist. Table 2. ROBUST criteria. Table 3. Sample search string. Table 4. Settings of the data driven priors for the primary outcome mortality and secondary outcome unfavourable neurology at 90-180 days. Table 5. GRADE assessment of certainty of evidence. Table 6. Sensitivity analyses using different effect size priors for the Bayesian meta-analysis. Figure 1. Frequentist meta-analysis of death and unfavourable neurological oucome. Figure 2. Sensitivity analysis for survival comparing initial shockable vs. non-shockable rhythms. Figure 3. Sensitivity analysis for neurological outcome comparing initial shockable vs. non-shockable rhythms. [file 13054_2022_3935_MOESM1_ESM.pdf]
